# The Effect of Intraradicular Multiple Fiber and Cast Posts on the Fracture Resistance of Endodontically Treated Teeth with Wide Root Canals

**DOI:** 10.1155/2018/1671498

**Published:** 2018-08-15

**Authors:** Satheesh B. Haralur, Maram Awdah Al Ahmari, Safeyah Abdurrahman AlQarni, Mashael Khaled Althobati

**Affiliations:** ^1^Department of Prosthodontics, College of Dentistry, King Khalid University, Saudi Arabia; ^2^College of Dentistry, King Khalid University, Saudi Arabia

## Abstract

**Introduction:**

The endodontically treated teeth (ETT) with thin remaining radicular dentin thickness are predisposed to fracture; hence it requires the diligent selection and the execution of endodontic post treatment. The objective of the study was to evaluate the reinforcing effect of both multiple fiber reinforced composite (FRC) and Ni-Cr cast metal posts at anterior and posterior regions.

**Material and Methods:**

Forty recently extracted root canal treated canine and single rooted premolar teeth were used for the study. They were randomly divided into four groups (n=10) as: Group 1, single FRC post; Group 2, multiple FRC posts; Group 3, single Ni-Cr metal post, Group 4, multiple Ni-Cr posts. The posts were cemented with self-adhesive resin cement and subsequently restored with full veneer metal crown. The compressive static load at 130^0^ for canine and 45^0^ for premolar was applied with the cross-head speed of 0.5mm/minute until the fracture. The obtained data was analyzed using the Kruskal–Wallis and Pairwise comparison tests with SPSS.

**Results:**

The results indicate that multiple FRC post restored canine had the maximum fracture load (1843.80±7.13 N), followed by cast multiple posts (1648.99±26.84 N), single fiber post (1623±40.31 N), and cast metal single post (1493±27.33 N). A similar trend was observed in premolar with higher max fracture load with multiple FRC posts at 1920.86±20.61 N and multiple cast metal posts at 1735.43±6.05 N.

**Conclusion:**

The restoration of ETT with larger canals by multiple FRC and metal posts provides substantially higher fracture resistance in comparison to wider single post.

## 1. Introduction

Restoration of endodontically treated teeth (ETT) is the integral part of restorative dental practice. The ETT is more susceptible to both biological and mechanical failure in comparison to vital teeth [1]. The principal etiological factor attributed to the increased risk is tooth structure loss during access cavity preparation, root canal shaping, post space preparation, and previous caries or restorations [2]. The root canal treatment procedures are reported to be accountable for 38% reduction in flexural strength [3]. The suitable coronal restoration is required to resume the function, restore aesthetics, and serve as an abutment in fixed or removable prosthesis. The adequate coronal restoration is also essential to prevent the penetration of microorganisms through the coronal end of the root canal [4]. ETT with large coronal tooth structure deficiency often requires the placement of endodontic post. The researchers are unambiguous in their opinion regarding the role of endodontic post, that is, to retain the coronal restoration.

The requirements of the endodontic post include the good fitting accuracy, biocompatibility, high tensile, and fatigue strength for favourable distribution of masticatory forces. The cast post are indicated in an ETT with substantial loss of coronal tooth structure, especially in noncircular canals. However, the use of prefabricated fiber reinforced composite (FRC) post is increasing in contemporary dental practice due to less clinical time, aesthetic colour, and easy retrievability. The additional advantage of FRC post is a compatible modulus of elasticity with dentin and consequent reduced predisposition to the root fracture. Though the endodontic post is employed to retain the coronal restoration, the presence of post itself predisposes the tooth to the root fracture. Hence proper selection of post and meticulous clinical procedure is critical in the success of post endodontic restorations. The researchers report that multiple factors like post length, diameter, remaining radicular dentin thickness, and post adaptation are vital in endurance of ETT. The major two reasons for endodontic post failures are debonding of post and root fracture. Researchers recommended [2] the longer poster for better stress distribution and improved root fracture resistance. The preservation of tooth structure is a main criterion during selection of post diameter. Stern et al. [5] suggested that the post width should not exceed 1/3rd of root diameter. Minimum of one mm sound dentin around post is strongly advocated by Halle et al. [6]. The tooth restored with wider diameter cast metal post and less remaining radicular dentin diameter offers least resistance to fracture.

In the routine clinical practice, clinicians encounter the ETT with large, flared root canals due to improper shaping, internal resorption, and retrieval procedure for broken instruments or post. The closely adapted endodontic post is known to improve the fracture resistance of tooth and retention of the post. Prefabricated FRC posts are not corresponding in shape to the root canal in its entire length. Hence, they are supplied along with calibrated, specialized drills to shape the canal and enhance the adaptation to the post. Additional shaping of a root canal to match the prefabricated FRC post shape further compromises root fracture resistance. The wider canals require the larger amount of luting cement to fill the void between the post and root canal. The lesser mechanical strength of luting cements, nonhomogenous cement layer, lead to compromised bond strength of the post. The reported polymerization shrinkage of composite resin cements ranging from 1.2% to 6% [7] will further aggravate the stress concentration and failure in bonding. The fabrication of larger metal cast post for these wider canals is daunting task for the dentist. The bulky impression material or direct patter resin leads to the distortion and subsequent ill-fitting cast post.

The multiple posts for restoration ETT have numerous advantages like improved adaptation and reduced luting cement volume. The procedure also reduces the necessity to remove the radicular dentin to adapt the prefabricated larger post. Less research data is available on feasibility of utilizing the multiple posts during restoration of the wider root canal; the existing reports are contradicting and inadequate [8, 9]. The biomechanical forces are different at an anterior and posterior region due to variation in magnitude, angulation of forces, and morphology of teeth. Hence this experimental, in vitro study was designed with the objective of evaluating the effect of multiple Ni-Cr cast and FRC post restorations on the fracture resistance of ETT with large root canals. The purpose of the study also included comparing the reinforcing effect of multiple post's restorations at anterior and posterior teeth regions.

## 2. Materials and Methods

The study proposal was reviewed and approved by an institutional research ethics committee of College of Dentistry, King Khalid University. Recently extracted and single rooted, forty canine and premolar teeth were collected from the patient donors. The collected sample teeth were extracted for periodontal or orthodontic reasons. The patients were informed about utilizing their teeth for research, and written consent was obtained. The teeth samples were evaluated under the stereomicroscope (Axio Zoom, Carl Zeiss Micro Imaging Inc., Thornwood, NY, USA) at X 5 magnification to eliminate the teeth with micro cracks.

The exclusion criteria for the teeth samples include the caries, fractures, previous endodontic treatment, and dental anomalies. The root morphology was confirmed with mesiodistal, buccolingual intraoral radiographs. The average mean root length of canines was 22 ± 1.84 mm and 15.78 ± 1.74 mm for premolar teeth.

All the teeth samples were sectioned 2 mm coronal to cementum-enamel junction. Adequate access cavity was prepared on all the teeth and working length set at one mm short of an apical foramen. Following crown down technique, the root canals were prepared, shaped, and widened until F5 size, up to the working length using nickel-titanium rotary system (ProTaper, Dentsply Maillefer, USA). The intervening 3% sodium hypochlorite solution was used as irrigation between changes of files. The root canals were obturated with ProTaper gutta-percha cones and a sealer (AH Plus, Dentsply Maillefer, USA). Two layers of the adhesive tape were applied over the root surfaces of all teeth samples. Subsequently, they were embedded vertically into the autopolymerizing polymethyl-methacrylate acrylic blocks (ProBase Cold, Ivoclar Vivadent, Schaan, Liechtenstein) with the help of a dental surveyor. Tooth structure of 2 mm coronal to CEJ was maintained above acrylic block during implanting them within the blocks.

The post space preparation was initiated with the sequential use of Gates-Glidden and Peeso reamers up to the size 5. The post space length for all the teeth samples was maintained at 18 mm for canine and 15 mm for mandibular premolar. The due care was observed to maintain a minimum of 5 mm gutta-percha obturation in the apical area. The post space was irrigated with 3% sodium hypochlorite followed by normal saline and dried thoroughly with paper points.

The teeth samples were randomly divided into the following groups with each group consisting of 10 samples:

  Group IA: restoration with single FRC post: canine
  Group IB: restoration with multiple FRC post: canine
  Group IC: restoration with single cast post: canine
  Group ID: restoration with multiple cast post: canine
  Group IIA: restoration with single FRC post: premolar
  Group IIB: restoration with multiple FRC post: premolar
  Group IIC: restoration with single cast post: premolar
  Group IID: restoration with multiple cast post: premolar.

Groups consisting of single post (Groups IA, II, A) received the 1.5 mm diameter conventional FRC post. The Groups I B and II B received two 0.8 FRC posts. The main post was placed up to the post space and one auxiliary post was placed beside the main post as apical as possible without undue pressure. The posts used were cleaned with alcohol swab and post space was thoroughly dried with paper points. The self-adhesive composite resin cement was used as the luting cement without any preconditioning of radicular dentin. The coronal extended part of the post was sectioned by maintaining 6 mm above the remaining tooth structure. The extended part of the post was sectioned after the polymerization of luting cement. The posterior composite was used to build up the core with uniform height of 6 mm and 6-degree taper. The tooth preparation was extended on to the remaining tooth structure with uniform 2 mm circumferential ferrule and 1 mm deep chamfer finish line. The ferrule height was calibrated with the help of a digital caliper (Mitutoyo, Tokyo, Japan). The taper of the core was standardized using parallel milling machine (Bravo, Mariotti, Forlì FC, Italy). The core was standardized for all the specimens including the cast post with the help of cellulite vacuum foil duplicated over the standard core.

The Groups IC and II C received the custom made single nickel-chromium cast post and core. The resin pattern (Duralay, GC EUROPE NV Leuven, Belgium) was used for the fabrication of the cast post with direct technique. The Groups ID and II D received two separate cast posts in the diameter of 0.8 mm; the post placement was followed as described for Group IB or IIB. The cast posts were sandblasted with aluminum oxide, cemented with glass-ionomer luting cement (GC Fuji I. Alsip, IL, USA).

**Figure 1:**
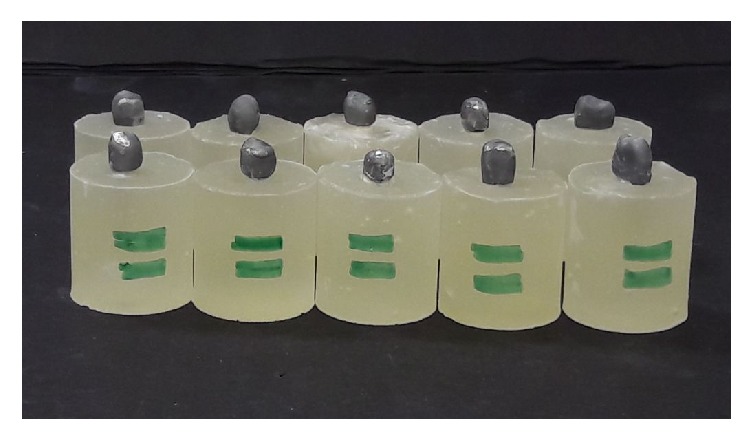
Teeth samples with metal coping embedded in resin blocks.

**Figure 2:**
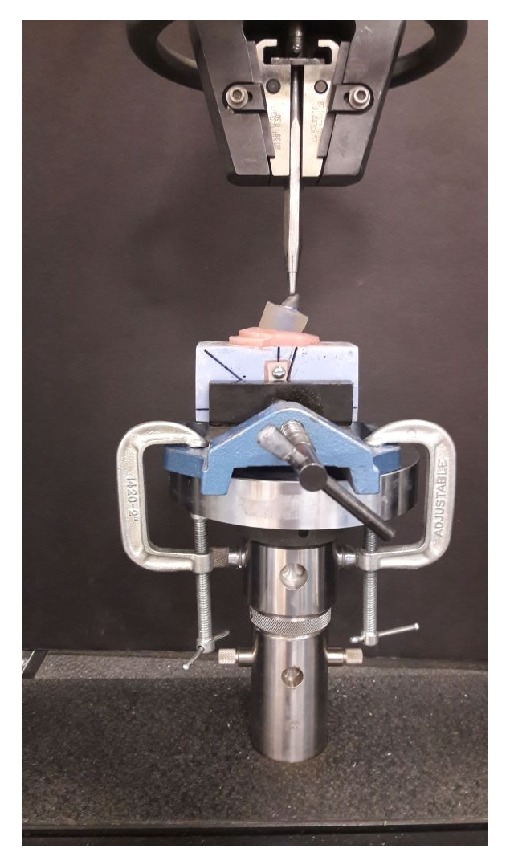
Teeth samples testing under universal testing machine.

The full veneer metal coping for the teeth samples was fabricated as per standard dental casting procedure using nickel-chromium alloys (Wiron 99, BEGO Bremer Goldschlägerei Wilh, Bremen, Germany) (Figure 1). The ledge was made at 2 mm from the incisal margin at the lingual surface of the anterior teeth metal coping. The ledge was useful for the uniform, stable loading. The metal castings were cemented using the type-I glass-ionomer luting cement (GC Fuji I. Alsip, IL, USA). The adhesive tape over the root surface on all the teeth samples was removed and the space gained within an acrylic block was relined with light body additional silicone impression material. The silicone was relined to simulate the cushioning effect of periodontal ligaments. The compressive force was applied on the lingual surface of the custom jig with 4 mm diameter round tip. The load was applied at 130 degrees to the long axis of the tooth for canine and 45 degrees to the longitudinal axis for the premolars (Figure 2). Static load at the cross-head speed of 0.5mm/minute was applied until the tooth fracture occurred and force at fracture was recorded.

The obtained data was statistically analyzed using the Kruskal–Wallis analysis and Pairwise comparison tests with SPSS 19 software (IBM Corporation, Armonk, New York, USA) to find the difference between the tested groups at 0.05 significance level.

## 3. Results

The mean and standard deviation (N) of the maximum load until failure for all the tested groups are presented in Table 1. The results from the study indicated that the maximum fracture load (N) for canine was recorded by ETT restored with multiple FRC posts (1843.80±7.13 N), followed by Ni-Cr cast multiple posts (1648.99±26.84 N), single FRC post (1623.98±40.31 N), and cast metal single post (1493.17±27.33 N). A similar trend was observed in premolar with higher fracture resistance, recorded by ETT restored with multiple FRC posts at 1920.86±20.61 N and cast metal multiple posts at 1735.43±6.05 N. The single FRC post groups recorded the fracture resistance of 1746.97±17.42 N and cast metal single post at 1629.00±6.08 N; both single post groups showed the least fracture resistance.

The Kruskal–Wallis was performed (Table 2) to assess significant differences between the groups. There was statistically significant difference in median values for canine groups with H value of 34.992 and p-value of 0.001. The mean rank was 16.50 for single FRC and 35.50 for multiple FRC posts. The cast metal single post had 5.60 mean rank; meanwhile it was 24.40 for cast metal multiple posts. The results also showed the statistically significant difference in premolar groups with H value of 34.723, p≤0.001, and mean ranks of 24.00, 35.50, 5.50, and 17.00 for respective groups.

Pairwise comparison between the groups (Table 3) showed the significant difference between all tested groups except the ETT restored with single FRC post V/S multiple metal posts in both canine and premolar teeth. The corresponding P-values between the groups were 0.131 and 0.181.

## 4. Discussion

Fracture of the post and the restored tooth is the most commonly reported failure of ETT restoration [10]. The remaining dentin thickness plays very critical role in fracture resistance of ETT [11]. The root canal treated teeth are hollow cylinder in shape; their strength is predominantly derived from the outer portions. The bending fracture resistance in annulus condition is proportional to the difference between the fourth powers of outer diameter and inner diameter radius [12]. Hence, the post does not reinforce the roots [13]. The restorative dentist regularly encounters the ETT with wider root canal requiring the restorations. The dentist should evolve the treatment plan to enhance the duration of clinical service by selecting an appropriate restorative method. The present study explored fracture resistance of ETT restored with multiple FRC and Ni-Cr metal posts in ETT with wider canals.

The study results showed the substantial increase in the fracture resistance of ETT restored with multiple posts in comparison to the single wider post. The outcomes were similar at different load's angle in posterior and anterior region. At the canine region the multiple FRC posts restored ETT had the fracture resistance of 1843.80 N, in comparison to 1623.98 N in single FRC post group. A similar tendency was observed in metal post with corresponding values of 1493.17 N and 1648.99 N, respectively. The researchers reported the existence of large difference in the stress produced and its distribution within the intact and post-core restored teeth [14]. The masticatory force initiates flexing stress similar to the short beam within intact natural teeth. The forces are distributed as the compressive stress on one side, tensile stress on converse side, and zero forces at the centre of cross section. The resultant forces are higher in compressive nature than tensile due to the shape, angulation of the tooth, and supporting alveolar bone. The stress delivery in ETT with post-core is noticeably dissimilar to intact teeth. It flexes as a single unit during mastication, and it leads to the increased tensile stress within the remaining tooth structure [15]. The multiple factors are attributed to the differences in stress distribution that include the stiffness, angulation of the post, and flexure of the remaining tooth structure [16, 17]. The larger difference between the flexure of the post and the remaining tooth structure leads to stress concentration and being prone to fracture [18]. The thin cross section of the remaining dentin thickness increases flexibility and susceptibility for fracture. The stiffness of the material is dependent on the cross-sectional area and elastic modulus and inversely proportional to the length of the element [19]. The multiple posts with small cross section area and similar length to the single wider post will have more modulus of elasticity in comparison to the single wide post. The multiple posts are held together with luting cement, and it adds to the flexibility of the post. The modulus of elasticity of resin cement is comparable to dentin and reinforced by forming inner tube with bonding to intraarticular dentin [20]. The multiple posts have more surface area than single large post; hence the stress between single post surface and the luting cement/dentin interface will be greater than multiple posts. The distribution of post in larger surface area helps to limit the crack formation by spreading the tensile stress in wider surface of luting cement/dentin. Maceri et al. [21] reported that the Von Mises stress is substantially reduced by 27% from intrusive load and 20% from oblique load with multiple post restorations. The Rankine stress to evaluate the risk of root fracture in multiple post solution also reduced the tensional stress at the apical and cervical region. The results of the study were in agreement with the findings of Q Li et al. [8] and Fráter et al. [9] that the multiple FRC posts achieved superior fracture resistance to teeth restored with single post.

The results of the present study indicated the better fracture resistance with FRC post in comparison to cast mental Ni-Cr posts in both configurations. The rigid post material is dissimilar to the pulp tissue as in vital intact teeth. The endodontic post with similar modulus of elasticity will assist in generating favourable stress-strain complex and simulate the mechanical behaviour of intact teeth. According to Pegoretti et al. [22]. FRC post displays the lowest peak stresses inside the root and induces a stress field comparable to that of natural tooth. The metallic posts are reported to induce the stress concentration at apical regions [23]. Barjau-Escribano et al. [24] reported the similar findings with FRC post having higher fracture resistance in comparison to metal post. The results of the study were in contradiction to the findings of Newman et al. [25] and Qing et al. [26], in which they reported higher fracture resistance in metal post. Few researchers like Hu et al. [27] and Fokkiga et al. [28] reported insignificant difference between the metal and FRC post fracture resistance. The difference in results could be due to the differences in methodology and testing procedures. Unlike most of the previous studies, the present study explored the fracture resistance in the teeth with wider root canal space. The fiber post with the resin luting cement is known to create the secondary monoblocks in the root canal; it reduces the stresses that occur inside the tooth structure and enhances the fracture resistance of the ETT [29]. Coelho et al. [30] conducted the finite-element analysis on the weakened roots and reported that the FRC were more durable and better at stress distribution.

The limitations of the study included the following: The static load was applied for testing samples, unlike masticatory forces in the mouth. The samples were not subjected to ageing and relatively small sample size. Further studies are required to evaluate the effect of different cements bonding to the radicular dentin and varied thickness of the luting cements on the fracture resistance.

## 5. Conclusion

Within the limitations of this in vitro study, ETT restored with multiple FRC posts yielded substantially higher fracture resistance than the single FRC restored teeth. The improved fracture resistance was observed in both anterior and posterior region. The statistically significant difference was recorded between the fracture resistance of multiple FRC posts and single FRC post at both areas. A similar trend of enhanced fracture resistance was observed in the multiple Ni-Cr metal posts in comparison to the single large metal post. The fracture resistance of FRC restored teeth was marginally higher than the metal post in both single and multiple configurations. Hence, results from the study indicate that the utilization of multiple posts in the weakened root canal provides better fracture resistance in both anterior and posterior regions.

## Figures and Tables

**Table 1:** Descriptive statistics of the fracture loads (N) measured in different groups.

Group	N	Mean (SD)	SD
**Canine**			
Fiber Post-Single	10	1623.98	40.31
Fiber Post-Multi	10	1843.80	7.13
Cast metal post-Single	10	1493.17	27.33
Cast metal post-Multi	10	1648.99	26.84

**Premolar**			
Fiber Post-Single	10	1746.97	17.42
Fiber Post-Multi	10	1920.86	20.61
Cast metal post-Single	10	1629.00	6.08
Cast metal post-Multi	10	1735.43	6.05

**Table 2:** Kruskal–Wallis analysis on maximum load measured in different groups.

**Tooth**	**Group**	**Mean Rank**	**Chi Square**	**df**	**P Value**
**Canine**	Fiber-single	16.50	34.992	3	0.001
	Fiber-multi	35.50			
	Cast metal-single	5.60			
	Cast Metal-multi	24.40			

**Premolar**	Fiber-single	24.00	34.723	3	0.001
	Fiber-multi	35.50			
	Cast metal-single	5.50			
	Cast Metal-multi	17.00			

Significance level is 0.05.

**Table 3:** Pairwise comparison between the maximum load recorded in different groups.

**Tooth**	**Group**	**Fiber** **Single**	**Fiber-** **Multi**	**Cast Metal-Single**	**Cast Metal-Multi**
Canine	Fiber-single	-	0.000	0.037	0.131
	Fiber-multi	0.000	-	0.000	0.034
	Cast metal-single	0.037	0.000	-	0.034
	Cast Metal-multi	0.131	0.034	0.034	-

Premolar	Fiber-single	-	0.028	0.000	0.181
	Fiber-multi	0.028	-	0.000	0.000
	Cast metal-single	0.000	0.000	-	0.028
	Cast Metal-multi	0.181	0.000	0.028	-

Significance level is 0.05.

## Data Availability

The data used to support the findings of this study are available from the corresponding author upon request.
